# Correlation between ^99m^Tc-TRODAT-1 SPECT and ^18^F-FDOPA PET in patients with Parkinson’s disease: a pilot study

**DOI:** 10.1590/0100-3984.2020.0087

**Published:** 2021

**Authors:** Julieta E. Arena, Leandro Urrutia, Germán Falasco, Magdalena Ponce de Leon, Silvia Vazquez, Malco Rossi, Marcelo Merello

**Affiliations:** 1 Fleni, Buenos Aires, Argentina.; 2 Pontificia Universidad Catolica Argentina, Buenos Aires, Argentina.

**Keywords:** Parkinson disease/diagnostic imaging, Parkinsonian disorders/diagnostic imaging, Tomography, emission-computed, single-photon/methods, Positron-emission tomography/methods, Tropanes/pharmacokinetics, Dihydroxyphenylalanine/analogs & derivatives, Doença de Parkinson/diagnóstico por imagem, Transtornos parkinsonianos/diagnóstico por imagem, Tomografia computadorizada de emissão de fóton único/métodos, Tomografia por emissão de pósitrons/métodos, Tropanos/farmacocinética, Di-hidroxifenilalanina/análogos & derivados

## Abstract

**Objective:**

To determine whether technetium-99m-labeled tropane derivative single-photon emission computed tomography (^99m^Tc-TRODAT-1 SPECT) provides results comparable to those of the less widely available, less accessible tool fluorine-18-labeled fluorodopa positron-emission tomography (^18^F-FDOPA PET) in the setting of a movement disorders clinic.

**Materials and Methods:**

In this prospective pilot study, eight subjects with a clinical diagnosis of Parkinson’s disease were randomly selected from among patients under treatment at a movement disorders clinic and submitted to ^99m^Tc-TRODAT-1 SPECT and ^18^F-FDOPA PET. The results were read by two experienced observers, and a semiquantitative analysis was performed.

**Results:**

The visual and semiquantitative analyses were concordant for all studies, showing that radiotracer uptake in the contralateral striatum on the most affected side was lower when ^99m^Tc-TRODAT-1 SPECT was employed. The semiquantitative analysis demonstrated a significant correlation between ^18^F-FDOPA PET and ^99m^Tc-TRODAT-1 SPECT (r = 0.73; *p* < 0.01).

**Conclusion:**

It appears that ^99m^Tc-TRODAT-1 SPECT is a valid option for the study of dopaminergic function in a clinical setting.

## Abstract (pt)

**Objetivo:**

Determinar se a ^99m^Tc-TRODAT-1 SPECT fornece resultados comparáveis aos da ^18^F-FDOPA PET, ferramenta menos acessível e menos amplamente disponível, no contexto de uma clínica de distúrbios do movimento.

**Materiais e Métodos:**

Neste estudo prospectivo, oito indivíduos com diagnóstico clínico de doença de Parkinson foram selecionados aleatoriamente entre pacientes em tratamento em uma clínica de distúrbios do movimento e submetidos a ^99m^Tc-TRODAT-1 SPECT e ^18^F-FDOPA PET. Os resultados foram lidos por dois observadores experientes e uma análise semiquantitativa foi realizada.

**Resultados:**

As análises visual e semiquantitativa foram concordantes para todos os estudos, mostrando que a captação do radiotraçador no estriado contralateral do lado mais afetado foi menor quando a ^99m^Tc-TRODAT-1 SPECT foi empregada. A análise semiquantitativa demonstrou uma correlação significativa entre ^18^F-FDOPA PET e ^99m^Tc-TRODAT-1 SPECT (r = 0,73; *p* < 0,01).

**Conclusão:**

A ^99m^Tc-TRODAT-1 SPECT parece ser uma opção válida para o estudo da função dopaminérgica em um ambiente clínico.

## INTRODUCTION

The main neurochemical hallmark of Parkinson’s disease (PD) is insufficient dopamine production by the substantia nigra pars compacta. Although PD is still diagnosed on the basis of clinical findings^(1)^, it is difficult to make the early differential diagnosis between true PD and other, nondegenerative forms of parkinsonism, such as those that are drug-induced, vascular, or even psychogenic, in which imaging studies could contribute to a more precise diagnosis^(2)^.

Several molecular imaging techniques capable of illustrating the different stages of dopamine synthesis, release, and uptake are currently available. Fluorine-18-labeled fluorodopa positron-emission tomography (^18^F-FDOPA PET) and dopamine transporter single-photon emission computed tomography (DAT-SPECT) have both been used for some time now, with well-established indications and diagnostic yields^(3,4)^. Although PET provides higher spatial resolution than does SPECT and is better for quantification purposes, SPECT is more affordable and more readily available in clinical practice as well as for epidemiological studies^(5,6)^.

The use of DAT-SPECT with ^123^I-ioflupane (DaTscan) has been approved by the US Food and Drug Administration (FDA) and European Medicines Agency (EMEA). In contrast, technetium-99m-labeled tropane derivative SPECT (^99m^Tc-TRODAT-1 SPECT) has not been approved for use in the United States or Europe, despite the fact that ^99m^Tc-TRODAT-1 has an effective half-life for imaging of 6.02 h and that ^99m^Tc-TRODAT-1 SPECT is extensively used in a number of other countries^(7-9)^. In addition, ^99m^Tc-TRODAT-1 is a biomarker of the DAT, which is found on the presynaptic membrane of dopaminergic projections and plays a role in extracellular dopamine regulation^(10)^. Several small studies have shown ^99m^Tc-TRODAT-1 SPECT to be a more affordable, accessible, kit-based imaging alternative that can effectively distinguish between normal and abnormal subjects^(11,12)^.

The aim of this study was to investigate the correlation between ^99m^Tc-TRODAT-1 SPECT and ^18^F-FDOPA PET.

## MATERIALS AND METHODS

### Subjects

Eight patients with a clinical diagnosis of PD, according to current criteria^(1)^, were recruited prospectively and randomly from among patients under treatment at a movement disorders clinic. To reduce the risk of misdiagnosis, we recruited only patients who had been diagnosed at least 2 years prior and had shown a sustained response to dopamine replacement therapy.

All of the patients underwent clinical examination by an experienced neurologist and scored according to the Hoehn and Yahr (H&Y) scale^(13)^ and the Movement Disorder Society-sponsored revision of the Unified Parkinson’s Disease Rating Scale (MDS-UPDRS) part III^(14)^ in the off state. Each subject underwent a ^99m^Tc-TRODAT-1 SPECT and an ^18^F-FDOPA PET, in random order.

The study protocol was approved by the local research ethics committee. All participating patients gave written informed consent.

### Imaging analysis

Patients were required to discontinue all dopaminergic medication overnight prior to both studies. For the ^99m^Tc-TRODAT-1 SPECT study, other potential test-altering medications, such as certain antidepressants, were also discontinued.

For the ^18^F-FDOPA PET examination, patients were premedicated with 200 mg of carbidopa (to inhibit peripheral L-dopa metabolism) 60 min before the administration of 370 MBq of ^18^F-FDOPA (ANMAT certificate no. 58050; Tecnonuclear SA, Buenos Aires, Argentina). At 120 min after tracer administration, images were acquired in a 64-slice PET scanner (Discovery 690; GE Healthcare, Waukesha, WI, USA). Patients were awake, in the supine position, with their head immobilized. Images were acquired with a static (30 min) protocol and were reconstructed using the ordered-subset expectation-maximization algorithm (2 iterations; 24 subsets). For the ^99m^Tc-TRODAT-1 SPECT examination, patients were injected with approximately 20 mCi of isotope DOPA-TEC (ANMAT certificate no. 57706; Tecnonuclear SA) and images were acquired 240 min later.

Visual and semiquantitative analysis was performed for ^99m^Tc-TRODAT-1 SPECT and ^18^F-FDOPA PET. Images were reconstructed by back-projection with a Butterworth filter at a cutoff frequency of 0.45, and the Chang method was employed for attenuation correction^(15)^.

For each pair of images from an individual patient, spatial co-registration was applied. The ^18^F-FDOPA PET images were spatially normalized to a reference ^18^F-FDOPA template^(16)^. Calculated transformation was applied to ^18^F-FDOPA PET and ^99m^Tc-TRODAT-1 SPECT images. Regions of interest (ROIs) for the striatum (caudate nucleus and putamen) were delineated in the ^18^F-FDOPA template and applied automatically to the ^18^F-FDOPA PET and ^99m^Tc-TRODAT-1 SPECT images. The ROIs in the occipital cortex were delineated and applied in a similar fashion to serve as reference areas. Specific uptake ratios (SURs) were calculated for each ROI and divided by the mean count in the occipital area, the SUR for each region thus being obtained. Asymmetry indices were calculated for the caudate nucleus and putamen through subtraction of the SUR on each side, divided by the mean values for both. The readers were blinded to the clinical data as well to previous individual PET or SPECT study results.

### Statistical analysis

The SURs obtained for the ROIs on ^99m^Tc-TRODAT-1 SPECT were correlated with those obtained on ^18^F-FDOPA PET. To compensate for the signal effects due to individual variation in specific uptake incorporation, we calculated the mean signal for each ROI pair (ipsilateral and contralateral to the most affected side). To evaluate the consistency between the two acquisition methods in terms of the difference between the ipsilateral and contralateral signals, linear regression was performed and Pearson’s correlation coefficient was calculated.

## RESULTS

The sample included three women and five men. The mean age was 58.3 ± 14.3 years (range, 35.8-74.9 years), and the mean disease duration was 34.3 ± 14.1 months (range, 15.1-57.1 months). One patient was classified as H&Y stage 1, whereas six were classified as H&Y stage 2 and one was classified as H&Y stage 3. The mean MDS-UPDRS part III (motor examination) score was 31.5 ± 14.2 (range, 10.0-53.0). All patients had asymmetric symptoms; one (subject 5) presented mild dyskinesias and motor fluctuations (Table 1). The mean time elapsed between the ^18^F-FDOPA PET and ^99m^Tc-TRODAT-1 SPECT examinations (or vice versa) was 3.6 ± 2.8 months (range, 0.4-8.3 months), and no treatment modifications were made during the interim.

**Table 1:** Demographic and clinical characteristics of patients with PD.

Subject	Gender	Age (years)	Clinical manifestation (most affected side)	Disease duration (months)	MDS-UPDRS (part III)	H&Y stage	Levodopa equivalent dose
1	Male	72.2	Akinetic-rigid (left)	51.9	43	3	750
2	Female	73.9	Akinetic-rigid (right)	25.0	27	2	300
3	Male	42.5	Tremor dominant (left)	36.3	37	2	750
4	Male	48.5	Tremor dominant (right)	24.1	10	1	160
5	Female	59.5	Akinetic-rigid (right)	57.1	38	2	1087
6	Male	61.2	Akinetic-rigid (right)	29.0	53	2	750
7	Female	72.4	Akinetic-rigid (left)	25.0	16	2	475
8	Male	55.8	Akinetic-rigid (left)	26.0	28	2	120

**Figure 1:**
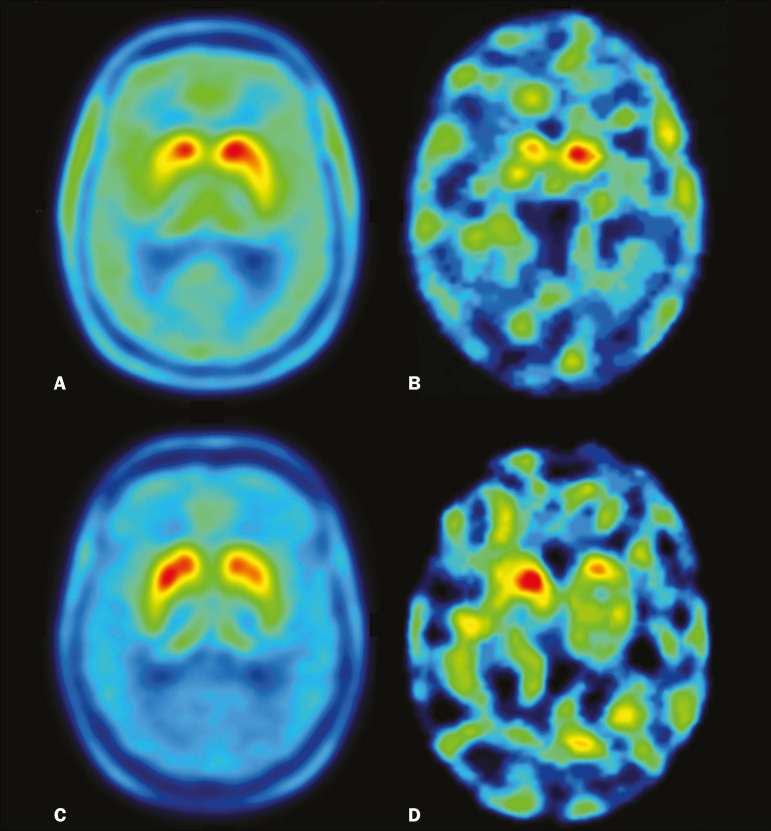
Representative transverse ^18^F-FDOPA PET image (left) and concordant ^99m^Tc-TRODAT-1 SPECT image (right) of patients with PD (**A,B:** subject 7; **C,D:** subject 6). **A,B:** Note the markedly low tracer uptake in the right putamen, the uptake also being low, to a lesser degree, in the left putamen. **C,D:** Inversely, tracer uptake was markedly low in the left putamen and low, to a lesser degree, in the right putamen.

For each patient, the visual comparison between the two methods was performed by two experienced observers and was concordant across all studies. Greater striatal signal loss was observed contralateral to the more affected side (Figure 1). As expected, the caudate nucleus and putamen were both better defined spatially and more readily identifiable on ^18^F-FDOPA PET images than on ^99m^Tc-TRODAT-1 SPECT images.

Semiquantitative analysis of the caudate nucleus and putamen ROIs showed greater loss of dopaminergic activity in the striatum on the contralateral side, and the degree of loss was comparable between the two techniques; except in subject 5, in whom, on ^99m^Tc-TRODAT-1 SPECT, the ipsilateral caudate nucleus was more affected, although the degree of asymmetry was only 3.4%, which was not significant (Table 2).

**Table 2:** SURs for the ROIs studied.

Subject	^18^F-FDOPA PET						^99m^Tc-TRODAT-1 SPECT				
	Putamen			Caudate nucleus			Putamen			Caudate nucleus	
	Contralateral	Ipsilateral		Contralateral	Ipsilateral		Contralateral	Ipsilateral		Contralateral	Ipsilateral
1	3.36	3.44		3.30	3.31		2.53	3.51		3.47	3.55
2	2.36	2.50		3.10	3.23		1.55	1.93		1.47	2.02
3	1.71	2.11		2.53	2.74		2.20	2.50		2.88	3.35
4	2.42	2.97		3.04	3.31		1.32	1.53		1.09	1.79
5	1.89	1.92		2.37	2.47		1.24	1.59		1.55	1.50
6	2.15	2.47		2.92	3.07		1.36	1.88		1.46	1.82
7	2.51	2.91		2.92	3.09		1.00	1.15		1.04	1.10
8	2.34	2.77		2.86	3.18		0.93	1.50		1.11	1.58

**Figure 2:**
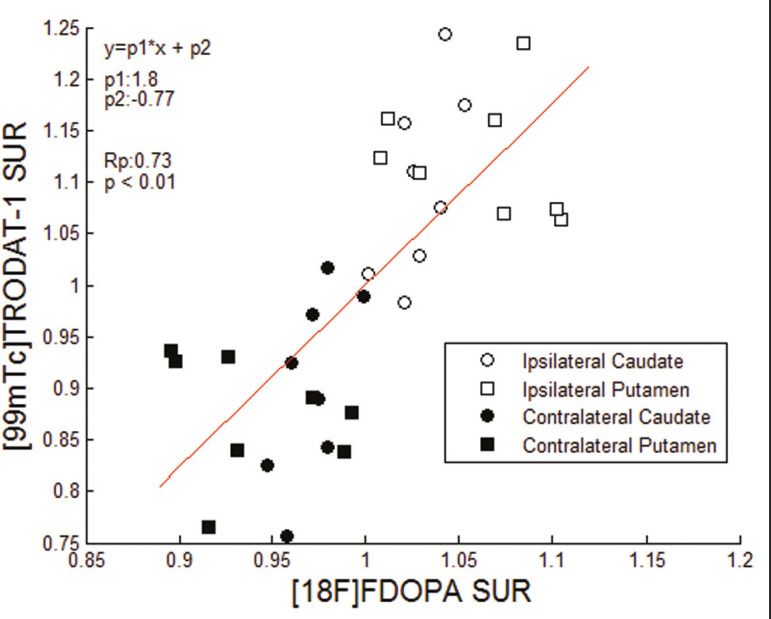
Correlation and linear regression of ^99m^Tc-TRODAT-1 and ^18^F-FDOPA uptake in ipsilateral and contralateral ROIs. Single points represent standardized SURs for each individual putamen and caudate nucleus.

**Figure 3:**
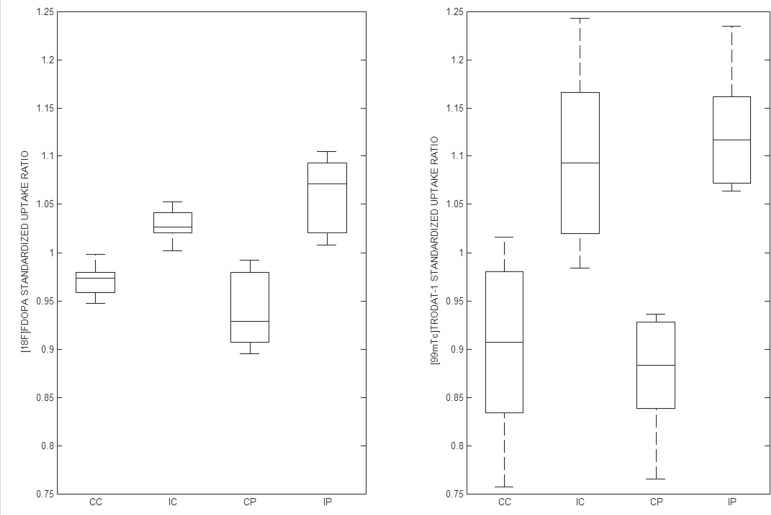
Box plot of standardized SUR group distribution for ^18^F-FDOPA (left) and ^99m^Tc-TRODAT-1 (right). CC: contralateral caudate nucleus; IC: ipsilateral caudate nucleus; CP: contralateral putamen; IP: ipsilateral putamen.

Taking all of the studies together, the semiquantitative analysis showed a statistically significant correlation between the two methods (r = 0.73; *p* < 0.01). The linear regression of predetermined ROIs (for the caudate nucleus and putamen, on the most affected and contralateral sides) is shown in Figure 2. The box plots in Figure 3 show that the differences between the ipsilateral and contralateral sides were greater on ^99m^Tc-TRODAT-1 SPECT images than on ^18^F-FDOPA PET images. The results of the visual and semiquantitative analyses were concordant across all studies, as well as being consistent with the clinical findings.

## DISCUSSION

In our sample of patients with mild to moderate PD, randomly selected from among patients under treatment at a movement disorders clinic, we found that ^99m^Tc-TRODAT-1 SPECT and ^18^F-FDOPA PET correlated well in relation to the visual and semiquantitative analyses. The ^18^F-FDOPA PET technique has become a well-established imaging modality for the study of such activity, providing good spatial resolution and allowing reliable quantification of dopaminergic activity, ultimately identifying the different parts of the striatum and quantifying levels of activity, which makes it very useful for research purposes. The ^18^F-FDOPA PET and ^99m^Tc-TRODAT-1 SPECT techniques differ with respect to the binding sites. Because ^18^F-FDOPA measures aromatic L-amino acid decarboxylase activity and ^99m^Tc-TRODAT-1 measures DAT availability, each is susceptible to different compensatory mechanisms. In ^99m^Tc-TRODAT-1 SPECT, compensation in early-stage PD consists in downregulation of presynaptic DATs in order to maintain high dopamine concentrations within the synapse. That increases the diagnostic sensitivity of this technique during the early stages of the disease.

To our knowledge, there has been only one study demonstrating that ^99m^Tc-TRODAT-1 SPECT has adequate sensitivity and specificity for the diagnosis of PD^(7)^. One previous study compared ^99m^Tc-TRODAT-1 SPECT and ^18^F-FDOPA PET in patients with PD in Taiwan^(17)^. However, there have been no such studies employing ^18^F-FDOPA PET as the gold-standard for evaluating dopaminergic function and comparing it with ^99m^Tc-TRODAT-1 SPECT in patients with PD in Latin America.

In the present study, the semiquantitative analysis showed that the variation within ROIs was greater for ^99m^Tc-TRODAT-1 SPECT than for ^18^F-FDOPA PET, likely because of the lower spatial resolution of SPECT, together with the nonspecific uptake of ^99m^Tc-TRODAT-1 and the higher counts obtained with PET, which would result in significant variations in quantification. In one instance (the caudate nucleus in subject 5), there was not even agreement between the quantitative and qualitative analyses. Therefore, we believe quantitative analysis in ^99m^Tc-TRODAT-1 SPECT should not be interpreted separately from a visual analysis by an experienced observer.

Our study has some limitations. Pathology specimens were not available to confirm PD in the individuals recruited, and the diagnosis was therefore established on the basis of the clinical findings alone, in accordance with currently accepted criteria^(1)^. In addition, because this was a pilot study, the number of subjects was limited. Nevertheless, the statistical power attained for the correlation analysis was acceptable.

Comparative studies have shown that SPECT techniques involving the use of tracers such as iodine-123-ß-CIT can be good alternatives to PET^(18,19)^. In 2011, the FDA approved the use of the DaTscan technique for the study of dopaminergic function, and the technique is now widely used. In contrast, the use of ^99m^Tc-TRODAT-1 SPECT has yet to be approved by the FDA or the EMEA. Nevertheless, in many countries, where ^99m^Tc-labeled tracers are more accessible and less expensive, ^99m^Tc-TRODAT-1 SPECT has become a widely available alternative in clinical practice.

## CONCLUSION

Given the accessibility and lower cost of ^99m^Tc-TRODAT-1 SPECT, together with the fact that it appears to correlate well with ^18^F-FDOPA PET, we believe that it should be considered a viable option for the imaging of patients with PD in daily practice.
